# A Turner syndrome case associated with dic(Y;22)

**DOI:** 10.1186/s13039-021-00556-z

**Published:** 2021-07-08

**Authors:** Rie Kawamura, Hidehito Inagaki, Midori Yamada, Fumihiko Suzuki, Yuki Naru, Hiroki Kurahashi

**Affiliations:** 1 Division of Molecular Genetics, Institute for Comprehensive Medical Science, Fujita Health University, 1-98 Dengakugakubo, Kutsukake-cho, Toyoake-shi, Aichi 470-1192 Japan; 2 Kariya Toyota General Hospital, Pediatrics, 5-15, Sumiyoshi-cho, Kariya-shi, Aichi 448-0000 Japan

**Keywords:** Telomeric associations, Turner syndrome, dic(Y,22)

## Abstract

**Background:**

Constitutional telomeric associations are very rare events and the mechanism underlying their development is not well understood.

**Case presentation:**

We here describe a female case of Turner syndrome with a 45,X,add(22)(p11.2)[25]/45,X[5]. We reconfirmed this karyotype by FISH analysis as 45,X,dic(Y;22)(p11.3;p11.2)[28]/45,X[2].ish dic(Y;22)(SRY+,DYZ1+). A possible mechanism underlying this mosaicism was a loss of dic(Y;22) followed by a monosomy rescue of chromosome 22. However, SNP microarray analysis revealed no loss of heterozygosity (LOH) in chromosome 22, although a mosaic pattern of LOH was clearly detectable at the pseudoautosomal regions of the sex chromosomes.

**Conclusions:**

Our results suggest that the separation of the dicentric chromosome at the junction resulted in a loss of chromosome Y without a loss of chromosome 22, leading to this patient’s unique mosaicism. Although telomere signals were not detected by FISH at the junction, it is likely that the original dic(Y;22) chromosome was generated by unstable telomeric associations. We propose a novel “pulled apart” mechanism as the process underlying this mosaicism.

**Supplementary Information:**

The online version contains supplementary material available at 10.1186/s13039-021-00556-z.

## Background

Telomeres are highly specialized structures that protect the ends of chromosomes and thereby prevent end-to-end fusions with other chromosomes. The loss of telomere function can lead to dicentric chromosomes as a result of end-to-end fusions, a phenomenon referred to as telomeric associations (TAs) [1]. Constitutional TAs are very rare events however [2–5]. Only about 10 cases of TAs between two autosomal chromosomes have been reported to date, all preferentially involving acrocentric chromosomes. Cases with TAs involving an autosome and chromosome Y have been occasionally reported. Cases of constitutional TAs between an autosome and chromosome Y have been generally reported as a mosaicism of two cell lines: a line with an end-to-end fusion of the Y and autosomal chromosomes, resulting in a dicentric chromosome, and a line whereby the dicentric chromosomes have been separated from each other and only the Y chromosome is lost [3, 5]. It is possible that the repeated TAs between an autosome and Y chromosome described in prior reports have been based on the recognizable Turner syndrome phenotype arising after the loss of the Y chromosome. The common feature of these cases is that the dicentric chromosomes are separated from each other, but the underlying mechanism of their development is not well understood. In our present case report, we present a detailed cytogenetic and genomic analysis of a female patient with Turner syndrome harboring a 45,X,dic(Y;22) and 45,X mosaicism and propose a novel “pulled apart” mechanism as the underlying process.

## Case presentation

Our current study patient was a girl of short stature with no notable external malformations other than a short neck. Cytogenetic analysis of her peripheral blood cells at the age of 13 revealed a 45,X,add(22)(p11.2)[25]/45,X[5]. She responded well to growth hormone therapy which commenced when she entered high school. After receiving approval from the Ethics Review Board for Human Genome Studies at Fujita Health University, and written informed consent from the patient’s parents, to allow her participation in our study, subsequent cytogenetic analyses were performed to identify the origin of the add(22) and gain insights into the underlying developmental mechanism. Since a Y chromosome was found in our patient, she underwent a gonadectomy to prevent gonadoblastoma. With regard to the patient’s internal genitalia, she had a hypoplastic uterus and organs that appeared to be ovaries and fallopian tubes at their expected location. Histopathologically, the ovaries and fallopian tubes were normal with no testicular tissue or tumor. She has been continued on estrogen therapy and is being followed as a female.

## Methods and results

We carried out G-band reanalysis and metaphase FISH analysis using peripheral blood cells from the patient (Additional file 1: Fig. 1). The FISH probes were specific for chromosome Y (SRY, DYZ1) (Cytocell, lnc., Cambridge, UK). By re-karyotyping using FISH analysis with an SRY probe, we confirmed the patient’s karyotype as mos 45,X,dic(Y;22)(p11.3;p11.2)[28]/45,X[2].ish dic(Y;22)(SRY+,DYZ1+). We increased the number of cells to 100 metaphases for G-banding and 200 interphase nuclei for FISH analyses and examined the mosaic ratio of two cell lines. It was concluded that the dic(Y;22) and 45,X cells represented 85–92% and 8–15%, respectively, of the population (Additional file 2: Fig. 2).

**Fig. 1:**
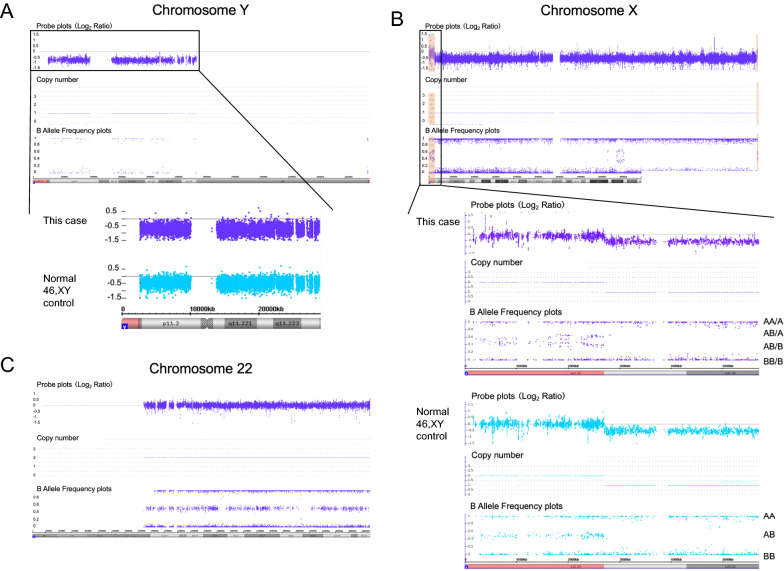
SNP microarray analysis. Probe plots, copy numbers, and B allele frequencies are shown for chromosome Y (**A**), chromosome X (**B**) and chromosome 22 (**C**). The lower panels in (**A**) and (**B**) are enlarged views for a comparison of the study patient (top) with a normal 46,XY control (bottom)

We also conducted SNP microarray analysis using a CytoScan HD Array (Affymetrix, Santa Clara, CA) to investigate how the patient’s mosaicism had been generated. Briefly, genomic DNA was extracted from her peripheral blood cells using a Gentra Puregene Blood kit (Qiagen, Hilden, Germany). Sample preparation was performed in accordance with the manufacturer’s protocol. The array scan data were visualized using ChAS 3.2 software (Affymetrix). The signals from the Y chromosome copy number probe were slightly lower than the normal 46,XY control, indicating a low level mosaicism of an entire Y chromosome loss (Fig. 1A). Accordingly, the SNP probes located in the pseudoautosomal region (PAR) revealed a mosaic pattern with 1 and 2 copies. In the normal 46,XY control, the B allele frequency (BAF) plots of PAR1 indicated AA, AB, BB SNP-genotypes. In our study patient however, the BAF of PAR1 showed an AA/A, AB/A, AB/B, BB/B mosaic pattern (Fig. 1B). In contrast, no copy number change or loss of heterozygosity (LOH) on chromosome 22 was detected (Fig. 1C). The BAF standard deviation for AB heterozygous probes on chromosome 22 was compared to other all autosomes and to the PAR on chromosome X. The standard deviation of the AB BAF plots on the PAR was 0.08, whereas that on all autosomes including chromosome 22 was 0.03 (Additional file 3: Fig. 3).

**Fig. 2:**
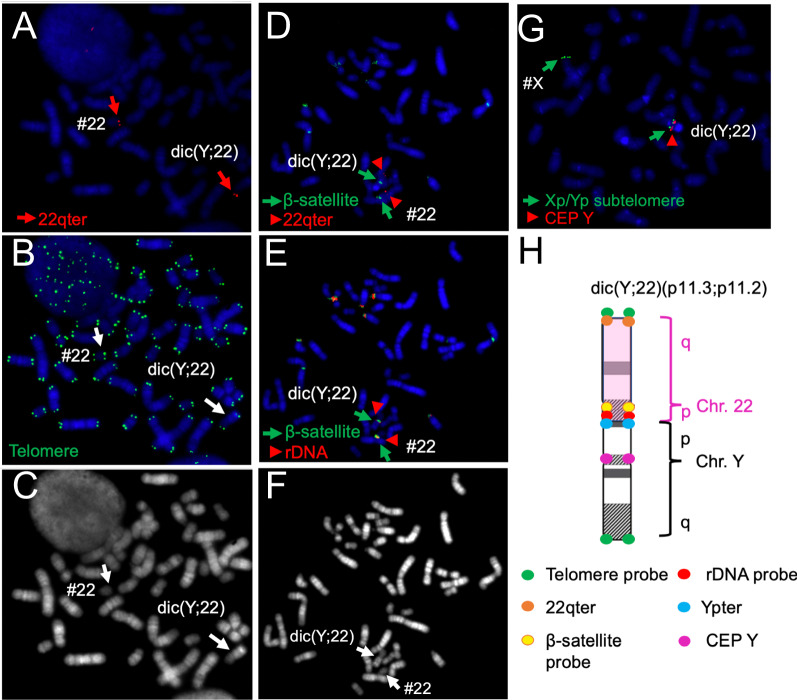
FISH analysis. The results shown in (**A-C**) are from the same metaphase as are those indicated in (**D-F**). (**A**) Probing of the subtelomeric region of chromosome 22q (red). (**B**) Probing of telomeric regions (green). The white arrows denote chromosome 22 and dic(Y;22). (**C**) FISH inverted DAPI image of (**A**). (**D**) Probing of the β-satellite region (green), and subtelomeric region of chromosome 22q (red). (**E**) Probing of the β-satellite region (green), and rDNA region (red). (**F**) FISH inverted DAPI image of (**E**). (**G**) Probing of the subtelomeric regions of chromosome Xp and Yp (green), and the centromeric region of chromosome Y (red). (**H**) Schema for the FISH analysis of dic(Y;22)

Based on our microarray data, we deemed that a loss of dic(Y;22) followed by a monosomy rescue of chromosome 22 was unlikely. Instead, we speculated that a separation of the dicentric chromosome at the junction resulted in the loss of chromosome Y without the loss of chromosome 22, leading to this patient’s unique mosaicism. Since it was possible that the original dic(Y;22) chromosome was generated by unstable TA, FISH was performed using telomeric and subtelomeric probes. As expected, chromosome 22 and chromosome Y were found to be joined between the short arms, and rDNA and β-satellite signals on 22pter, and a Ypter signal were observed between each centromere of dic(Y;22). However, no telomere signal was observed (Fig. 2). On both of the chromosomes 22 in the 45,X cell line, rDNA and β-satellite signals were confirmed, whereas signals originating from the Y chromosome were not observed.

## Discussion

We here report a patient with possible TA between chromosomes Y and 22 manifesting a Turner syndrome phenotype. Four cases with TAs involving an autosome and chromosome Y have been reported to date (Table 1) [3–5, 13]. All of these prior cases involve a mosaicism of cell lines with TA and with 45,X, suggesting the instability of the TA chromosome, resulting in a Y chromosome loss. Notably, two previous cases showed a jumping translocation-like karyotype, in which two independent cell lines were produced, each carrying TA between the Y chromosome and different autosomes. One case showed tas(Y;8) and tas(Y;16) in the peripheral blood, whereas the other harbored tas(Y;21) in peripheral blood but tas(Y;14) was identified in the gonadal tissue in addition to tas(Y;21) [4, 13]. These observations also suggested the instability of TA chromosomes.

**Table 1:** Summary of previously published TA cases

Chromosome abnormality in the blood	Cell proportions (%)	Chromosome loss in 45,X	Clinical features	References
45,X/46,X,tas(Y;7)	3/97	Y	Gonadal dysgenesis	Beneteau et al. [3]
45,X/46,X,tas(Y;16)/46,X,tas(Y;8)	40/40/20	Y	Ambiguous genitalia	Zhang et al. [4]
45,X/46,X,tas(Y;19)	33/67	Y	Premature ovarian insufficiency	Barnabas et al. [5]
45,X/46,X,tas(Y;21)*	50/50	Y	Turner syndrome	Sawyer et al. [13]
45,X/45,X,dic(Y;22)	8/92	Y	Turner syndrome	Present case

*The left gonadal tissue of this patient showed an evolution of 2 additional cell lines i.e. 45,X,tas(Y;21)(q12;p13),−22/46,X,tas(Y;21)(q12;p13),+tas(Y;14)(q12;p13),−22

**Fig. 3:**
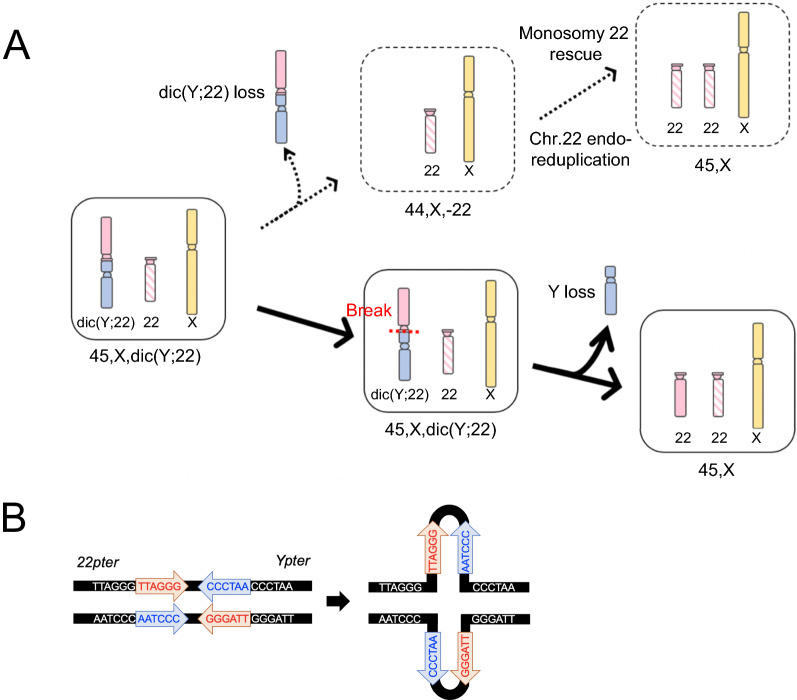
Possible mechanisms underlying the mosaicism in the study patient. (**A**) One hypothesis for the mechanism underlying the study patient’s karyotype is that a dic(Y;22) loss was followed by endo-reduplication of the remaining normal chromosome 22 (upper diagram). Alternatively, dic(Y;22) may have undergone a break at the junction followed by loss of the Y segment and telomere healing at 22p (lower diagram). (**B**) Predicted secondary structure following end-to-end telomere fusion. The resulting palindromic sequences have the potential to form a cruciform structure via intrastrand-base pairing of single-stranded DNA. The sequences indicated by red arrows are complementary to those indicated by blue arrows

There were two possible developmental mechanisms for the mosaic karyotypes in our current study case harboring a 45,X,dic(Y;22)/45,X karyotype. One was a loss of dic(Y;22) followed by a monosomy rescue of chromosome 22 (Fig. 3A, top), and the other was a dic(Y;22) separation at the junction followed by a loss of chromosome Y (Fig. 3A, bottom). We concluded that endoduplication of chromosome 22 was unlikely in our current case since the SNP microarray results showed no evidence of low LOH levels on chromosome 22. These data strongly suggested instead that chromosomes Y and 22 were separated at the junction of dic(Y;22) and that chromosome Y was lost shortly after that breakage took place (Fig. 3A, bottom). Thus, we speculated from our current findings for this patient that the original dic(Y;22) chromosome was generated by unstable TAs. Since similar SNP microarray data were also previously reported for tas(Y;7), the mosaicism in our present patient is likely to have arisen via the latter process [3]. We hereby propose a novel “pulled apart” mechanism which is caused by TA fragility and that leads to this unique mosaicism.

Exposed telomere ends are generally recognized as DNA breaks by the DNA repair system, and thereby joined to other DNA breaks by end-to-end fusion via non-homologous end joining (NHEJ). If the telomeres are sufficiently long however, the DNA ends may form a secondary structure known as a telomere T-loop which will be shielded from a DNA repair reaction with the aid of a protein complex called shelterin. TAs can take place if this protection is impaired. The telomeres shorten with each successive chromosome replication and cell division cycle [6, 7]. When this shortening reaches a certain point with aging, the protective response from T-loop formation can become less effective and TAs can be generated. Thus, the telomere sequence at the junction of a TA may be too short to be detectable by telomere FISH. Notably in this regard, some reported TA cases show positive telomere FISH signals while others do not [3, 5].

A still unresolved question with regard to our current case is why the breakage occurred at the junction and the two chromosomes were separated again. When telomeres recombine with each other through NHEJ, they are predicted to form inverted repeats, i.e. palindromic sequences, which may then form a cruciform structure (Fig. 3B). Such palindromic sequences have been often identified at the breakpoints of constitutional translocations in humans, the best studied example of which is t(11;22) [8, 9]. Such an unstable DNA secondary structure would be a target for DNA cleavage enzymes, thus leading to the recurrent translocation [9]. In this context, TA cases may always have a risk of secondary rearrangements, including jumping translocations [10].

A considerable subset of Turner syndrome cases develop as a 45,X mosaicism [11, 12]. Previously reported cases involving TAs between the Y chromosome and the autosomes manifested a phenotype involving a mixed gonadal dysgenesis due to Y chromosome loss (Table 1) [3–5, 13]. Since the mosaic ratio varies among patients, and also varies from tissue to tissue, some cases manifested mixed gonadal dysgenesis and others manifested a Turner phenotype. It is well documented that the co-existence of cells with and without a Y chromosome in the same gonads indicates a high risk of gonadoblastoma. Since there is a high risk of gonadoblastoma in Turner syndrome patients involving the Y chromosome [14], our current patient underwent a gonadectomy and fortunately no evidence of gonadoblastoma was detected. Turner syndrome cases carrying a mosaicism due to TAs between the Y chromosome and autosome should always receive preventative treatment for possible gonadoblastoma.

## Conclusions

The mechanism underlying the development of the 45,X,dic(Y;22) and 45,X mosaicism identified in our present Turner syndrome patient may have been due to the fragility caused by TAs. If we analyze the structure around the junction at the sequence level using a long-read sequencer, we might in the future be able to elucidate the mechanism of TA development in more detail.

## Supplementary Information

**Additional file 1.** Fig. 1 G-banding analysis of the study patient

**Additional file 2.** Fig. 2 Mosaic ratio results

**Additional file 3.** Fig. 3 BAF standard deviation for AB heterozygous probes

## Data Availability

Please contact the corresponding author regarding any data requests.

## Abbreviations

FISH: fluorescence in situ hybridization
LOH: loss of heterozygosity
TAs: telomeric associations
SNP microarray: single nucleotide polymorphism microarray
PAR: pseudoautosomal region
BAF: B allele frequency
NHEJ: non-homologous end joining
