# Supplementary material for: A Turner syndrome case associated with dic(Y;22)
Source: Mol Cytogenet. 2021 Jul 8;14:34. doi: 10.1186/s13039-021-00556-z (PMC8264959; doi:10.1186/s13039-021-00556-z)
Supplement: Supplementary file 1 — Additional file 1. Fig. 1 G-banding analysis of the study patient [file 13039_2021_556_MOESM1_ESM.pptx]

## Slide 1
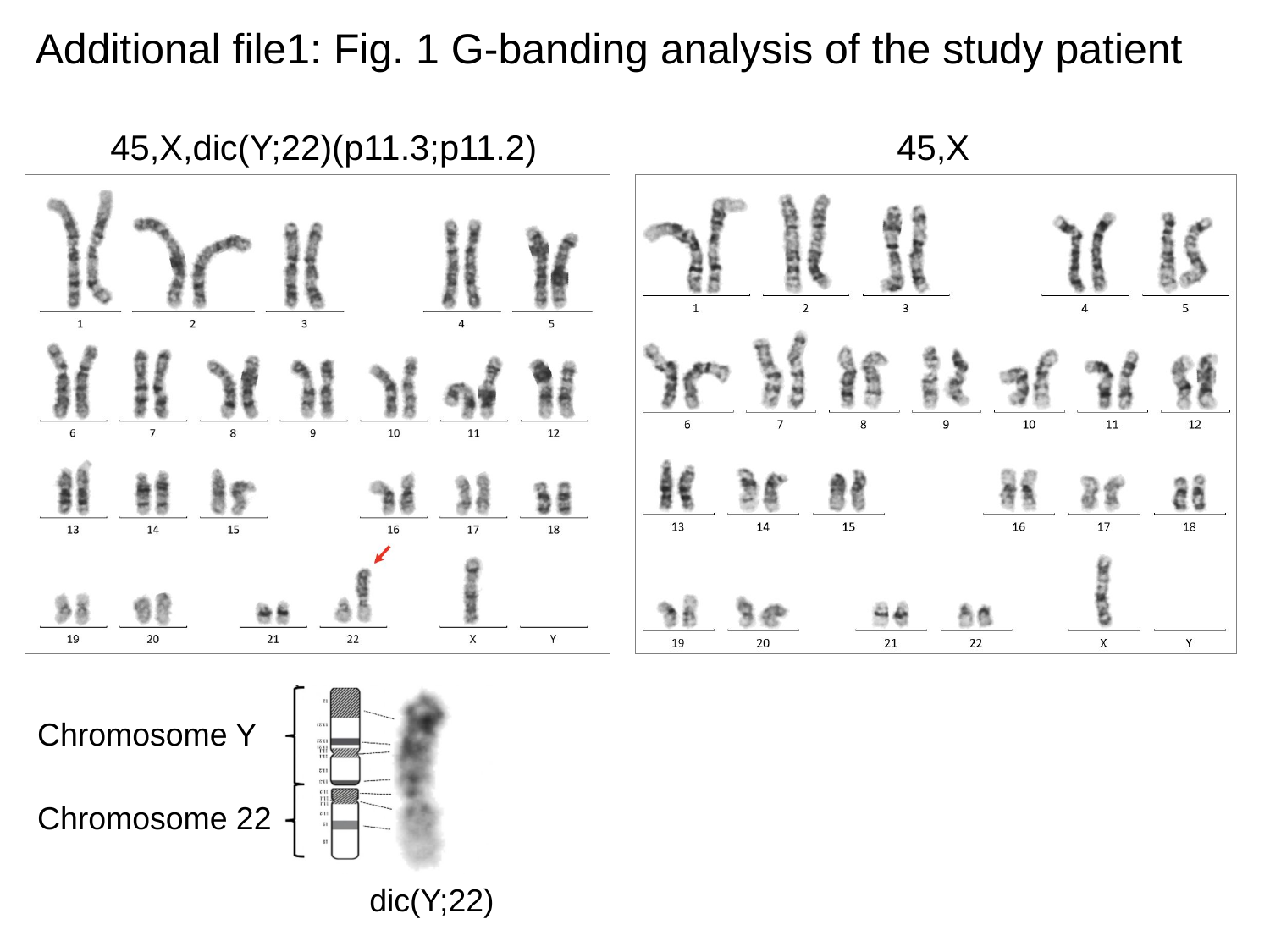

Additional file1: Fig. 1 G-banding analysis of the study patient
45,X,dic(Y;22)(p11.3;p11.2)
45,X
Chromosome Y
Chromosome 22
 dic(Y;22)
