# Supplementary material for: A Turner syndrome case associated with dic(Y;22)
Source: Mol Cytogenet. 2021 Jul 8;14:34. doi: 10.1186/s13039-021-00556-z (PMC8264959; doi:10.1186/s13039-021-00556-z)
Supplement: Supplementary file 2 — Additional file 2. Fig. 2 Mosaic ratio results [file 13039_2021_556_MOESM2_ESM.pptx]

## Slide 1
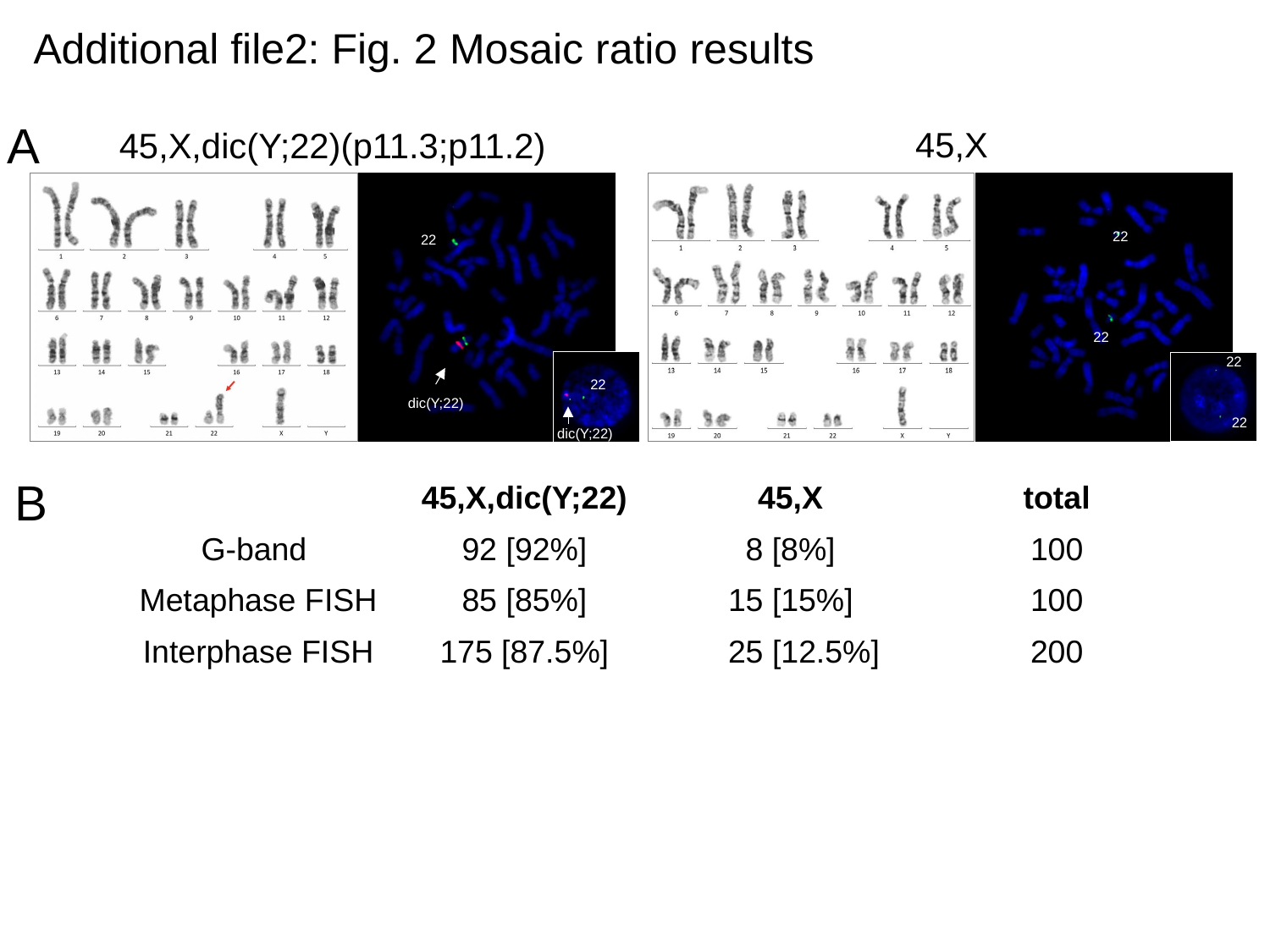

Additional file2: Fig. 2 Mosaic ratio results
A
45,X
45,X,dic(Y;22)(p11.3;p11.2)
22
22
22
22
22
dic(Y;22)
22
dic(Y;22)
B
| | 45,X,dic(Y;22) | 45,X | total |
| --- | --- | --- | --- |
| G-band | 92 [92%] | 8 [8%] | 100 |
| Metaphase FISH | 85 [85%] | 15 [15%] | 100 |
| Interphase FISH | 175 [87.5%] | 25 [12.5%] | 200 |
