# Supplementary figures and images for: A Turner syndrome case associated with dic(Y;22)
Source: Mol Cytogenet. 2021 Jul 8;14:34. doi: 10.1186/s13039-021-00556-z (PMC8264959; doi:10.1186/s13039-021-00556-z)

## Slide 1
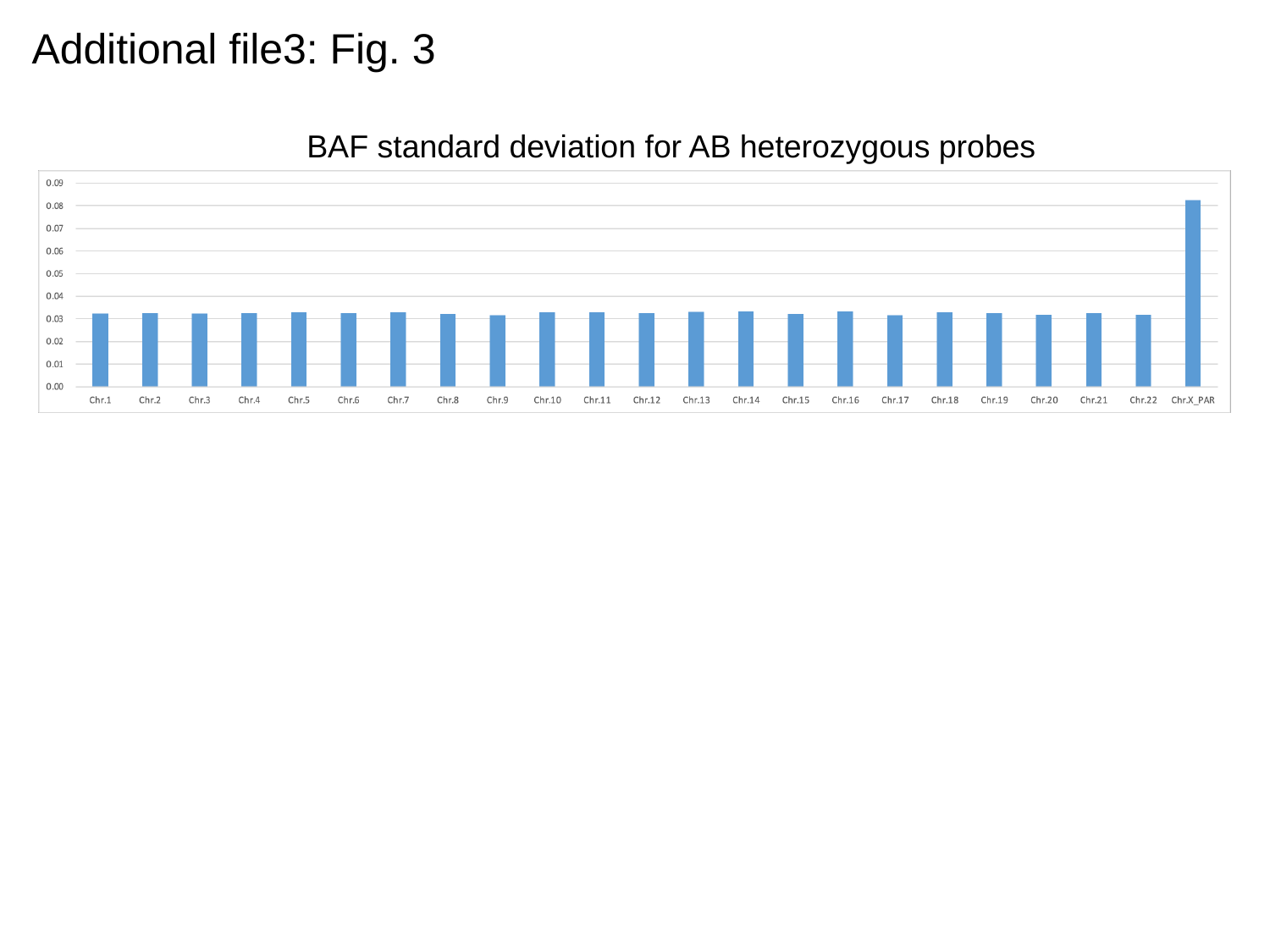

Additional file3: Fig. 3
BAF standard deviation for AB heterozygous probes

Supplement: Supplementary file 3 — Additional file 3. Fig. 3 BAF standard deviation for AB heterozygous probes [file 13039_2021_556_MOESM3_ESM.pptx]
